# Burden of care and coping strategies among informal caregivers of people with behavioral and psychological symptoms of dementia in rural south-western Uganda

**DOI:** 10.1186/s12877-023-04129-0

**Published:** 2023-08-08

**Authors:** Judith Owokuhaisa, Ronald Kamoga, Pius Musinguzi, Moses Muwanguzi, Sylivia Natukunda, Vincent Mubangizi, Keith Asiime, Godfrey Zari Rukundo

**Affiliations:** 1 Mbarara University of Science and Technology, Mbarara, Uganda; 2 Department of Psychiatry, Mbarara University of Science and Technology, PO Box 1410, Mbarara, Uganda

**Keywords:** Caregiver, Burden, Coping strategies, Behavioral and psychological symptoms of Dementia

## Abstract

**Background:**

Caregiving is a draining role that inflicts a significant level of burden upon caregivers for older people with Behavioral and Psychological Symptoms of Dementia (BPSD). Caregiver burden is associated with poor health outcomes for both the people with BPSD and their caregivers. This study explored the burden of care and coping strategies used by informal caregivers of older people with BPSD in rural Southwestern Uganda.

**Methods:**

This was a qualitative study among informal caregivers of older people with BPSD in Rubanda and Rukiga districts. We conducted in-depth interviews with a purposive sample of 27 caregivers using an interview guide. The interviews were conducted in the local language, audio recorded, transcribed, translated into English, and thematically analyzed.

**Results:**

There were two major themes: caregiver burden and coping strategies. Caregiver burden was described as financial, physical, psychological and social. Caregivers mainly used emotion-focused coping strategies (religious coping, acceptance and emotional support seeking). Problem-focused coping strategies (planning) and dysfunctional coping strategies (self-distraction) were used to a lesser extent.

**Conclusion:**

Informal caregivers of people with BPSD adopted both emotional and problem-focused coping strategies to cope with the burden of care for people with BPSD. Such coping strategies seemed to lighten the burden of caring, in the long motivating the caregivers to continue with the caring role.

## Background

Alzheimer’s disease and related dementias (ADRD) affected over 55 million people worldwide in 2020. According to the World Health Organization (WHO) report of 2019 the number of people with dementia is expected to double every 20 years. The majority of people with ADRD live in low and middle-income countries, [1]. In sub-Saharan Africa, the dementia burden is expected to rise by approximately 300% from 1.69 million people in 2015 to 5.72 million in 2050 [2]. Information about dementia and related disorders in Uganda is scanty. A recent Ugandan study among people aged 60 years and above reported a dementia prevalence of 20% [3], about three times higher than the estimated prevalence of 7.2% in Sub-Saharan Africa [4]. According to previous research, about 90% of people with dementia develop one or more of the Behavioral and Psychological Symptoms of Dementia (BPSD) [5]. BPSD are known as neuropsychiatric symptoms that represent a heterogeneous group of non-cognitive symptoms occurring in individuals with dementia. These include anxiety, agitation, aberrant motor behavior, irritability, depression, disinhibition, hallucinations and sleep changes [5]. Importantly, 50% of patients simultaneously have at least four neuropsychiatric symptoms [6]. BPSD symptoms are associated with a considerable amount of caregiver stress, depression and anxiety related to prolonged hospitalizations and employment disruptions [7, 8].

Caring for people with BPSD requires considerable effort and adaptive behaviors from their caregivers. While performing the caregiving role, caregivers encounter significant caregiver burdens. This is the stress, tension, and anxiety that caregivers experience when they are faced with challenges when caring for the care receiver [9]. BPSD exerts huge negative consequences on the caregivers, and family support systems. The burden is worsened by several factors including disparities in access to health services [10]. Caregiver burden in form of economic constraints is even worse in low and middle-income countries. In such settings, the caregiver families are overwhelmingly susceptible to the negative economic impact of caregiving [11]. In addition, challenging behaviors (agitation and wandering) and the safety of older people with BPSD are a source of distress to the caregivers [12]. Previous studies have shown caregiver burden to immensely affect caregivers’ physical and mental well-being which in turn compromises the quality of care given to people with BPSD [9]. In addition, mental health problems like anxiety and depression have been reported among caregivers of patients with dementia, which compromises the emotional and physical care offered to older people with dementia [13].

In response to the distress derived from the caregiving burden, caregivers used various coping strategies to ameliorate the burden and its effects [14]. Coping strategies have been categorized into three that is task (problem) -focused, emotion-focused, and avoidance-focused (dysfunctional) strategies [15]. In emotion-focused coping a person tries to minimize the negative emotional outcomes of stress. On the other hand, problem-focused coping aims at resolving the stressful situation or removing the source of stress [16]. Dysfunctional coping strategies involve behavioural disengagement, denial, self-distraction, self-blame and substance use [17]. Several factors (such as educational level, cognitive ability, life experience, social skills, social support, self-esteem and personality) have been associated with the coping strategies used by caregivers [18]. This study aimed to explore the caregiver burden and coping strategies among caregivers of people with BPSD in rural southwestern Uganda.

## Methods

### Study design

This was a cross-sectional qualitative study that explored the caregiver burden and coping strategies using in-depth interviews.

### Study setting

We conducted the study in two districts of Kigezi sub-region (Rukiga and Rubanda districts) in southwestern Uganda, where most of the population depends on small-scale subsistence farming. The two districts are estimated to have 100,726 and 196,896 people respectively [19]. The Kigezi sub-region has about 6% of all old people (60 years and above) in Uganda, which makes it the sub-region with the highest share of older people, compared to all other sub-regions of Uganda. Old people in this region have been estimated to live to attain the highest average age of 71 years in comparison to other regions of the country such as Karamoja sub-region at 69 years, Busoga at 70 years and Kampala at 69.7 years [20]. With an increase in life expectancy in Uganda, more older people in this sub-region are expected to contribute to a larger portion of the population with dementia in Uganda [20].

### Study population and sampling

Using purposive sampling, we selected twenty-seven informal caregivers of people living with dementia with the help of a representative of the Reach One Touch One Ministries (ROTOM) in Rukiga and the village health teams (VHTs) in Rubanda district. ROTOM is a Christian organization taking care of older people and children [21]. Village Health Teams (VHTs) are volunteer community health workers selected from their communities, as the first contact with the health system. They help to promote health at household and community levels and link the population to health facilities (Uganda MOH, 2010). We enrolled study participants until saturation was achieved. Saturation was reached by consensus within the team at the point when no new emerging themes were obtained with successive interviews.

### Data collection tools

We used an interview guide with probes to get the detailed information regarding distress faced by caregivers of patients with BPSD and how they cope with it. We conducted 27 in-depth interviews using open-ended questions aligned with the study objectives. Here below are some of the guiding questions that were asked. (1) Please describe in as much detail as possible your experience being a caregiver (Probe: Could you please tell me what it is like to be a caregiver?) (2) How has caregiving affected your life? (3) Can you describe your caregiving on an ordinary day? (4) What support have you received in your role as a caregiver? (5) Since you have been taking care of this person, how stressful has your life been? How difficulty had it been to be a caregiver? (6) What has been difficult? (7) How have you been caring for yourself? What has been difficulty in caring for yourself? (8) What has been difficult with family members or other close persons? (9) What help have you needed to care for yourself, family, or person with BPSD? (10) How would you describe the effect your relative/ person’s behavior and symptoms had on your ability to care for her/him? (11) Some caregivers feel they can deal with their situation, other caregivers feel they are failing; what is it like for you? (12) How do you see your ability to cope in the future? (13) What helps you to cope better with your situation? (14) Have there been moments when you thought, you just didn’t know what to do anymore? If yes, what did you do in such situation? (15) What events gave you the feeling that you couldn’t manage? (16) Who or what supported you in such situation?

### Data collection procedure

The lead author and three trained research assistants conducted the in-depth interviews in June 2022. The interviews were conducted in a noise-free and private place. Each interview lasted approximately 40 min and they were conducted in Runyankore-Rukiga, the local language. All interviews were audio recorded and supplemented with field notes. At the end of the interview, participants received a token of appreciation of 10,000 Ugandan Shillings (approximately US$ 2.86 at the time of the study).

### Quality control

The lead author and all research assistants were trained on study objectives, BPSD, ethical conduct of research and data collection procedures. Interview guides were tested for clarity and usability in a separate community before data collection inception. There were feedback meetings at the end of each day of data collection to check for the completeness of the data.

### Data management and analysis

Inductive and deductive content analysis was used. Data were transcribed verbatim by research assistants and compared with the audio recordings to verify the correctness and consistency of transcripts. The transcripts in Ruyankore-Rukiga were then translated into English by an independent translator who is fluent in local languages with a social science background and enriched with qualitative data translation skills. Two authors (JO and PM) independently read through the transcripts and developed codes and categories based on themes that emerged from the content and then the codes were discussed for consensus. A codebook was then developed and used to complete the coding. Two main themes emerged from our results: Caregiving burden and coping strategies.

## Results

### Demographic characteristics of study participants

Of the 27 participants, 12 were males and 15 were females; 25 were married and 2 were widowed (Table 1).

**Table 1:** Demographic characteristics of participants (N = 27)

Caregiver variable/characteristics	Frequency (%)
Age	
31–40	4(14.8)
41–50	8(29.6)
51–60	9(33.3)
> 60	6(22.2)
**Gender**	
Male	12(44.4)
Female	15(55.5)
**Marital status**	
Married	25(92.6)
Widow	2(7.7)
**Relationship with the older person**	
Mother	11(40.7)
Father	2(7.7)
Grandparent	1(3.7)
Husband	5(18.5)
Mother-in-law	3(11.1)
Sister-in-law	1(3.7)
Aunt	1(3.7)
Brother	1(3.7)

Two major themes that are caregiver burden and coping strategies emerged. Several sub-themes were developed under each major theme. (Table 2)

**Table 2:** Themes and sub-themes

Theme	Sub-theme	Categories
**Caregiver burden**	Financial burden Physical burden Psychological burden Social burden	
**Coping strategies**	Religious coping acceptance Social and emotional support seeking	Emotion-focused coping
	Planning Persistence Believing in oneself	Problem-focused coping
	Self-distraction	Dysfunctional coping

### Caregiver burden

The caregivers of older people with BPSD mentioned various sources of burden when performing their daily care roles. The sources of burden were financial burden, physical burden, psychological burden, and social burden.

#### Financial burden

The caregivers shared that they spent a lot of money when caring for older people with BPSD which was causing a significant burden on them. They mentioned that they spent money to buy food, medicine, clothes and pay transport charges to health facilities for older people with BPSD. More so, the caregivers revealed that older people with BPSD can no longer eat readily available food or some foods are restricted due to health reasons. This in turn leaves the caregivers with no option other than using the little money they have to buy the recommended food. The caregivers further mentioned that the financial burden was worsened by a lack of time to do income-generating activities.

“*…Another thing how it affects me in my life; is economically, I spend a lot. You find that my income is small yet the expenditure is very high. So, you find that sometimes I don’t have the money and yet am supposed to take care of her, buy for her a good dress, clothing, am supposed to give her shoes, am supposed to employ someone to stay with her”***(40 years old, married female).**

**“***We don’t have land to dig and getting food Is hard, even what to wear is not there so I am the one who tries to get her clothes so that she does not go naked and the money is not always there” (participant cries)***(46 years old, widowed female**)

Some participants shared that they could no longer meet the financial demands of their own families like paying school fees for their children. For instance, one caregiver said that she took her granddaughter out of school to use the money for school fees to take care of her father. Some caregivers said that they had resorted to selling family property like land to raise money. Another caregiver mentioned that he had started borrowing money from friends and local groups to raise money to take care of the patient.

*“Like that girl, I told you about she should be in school but she dropped out to help me take care of her father, even my son in the army who was catering for her education told her to first wait and we use her school fees money to take care of their father at least for a year*” **(48 years old, married female).**

*“Failure to get money, and also when the child is sent out of school and suffering when I am, alone….Failure to get money to pay in the hospital I even sold my piece of land and I paid the debts”****(50 years*old, married female*).***

#### Physical burden

The caregivers stated that caregiving affects their physical well-being. Caregivers described feeling tired, spending sleepless nights or even falling sick. The caregivers mentioned how some caregiving roles were tiring while caring for the older person. The caregivers also had their own families to take care of. Yet, the caregivers were not being assisted by other family members.

“*What disturbs me, is sometimes whenever I work very much, I have my family I have the other family and when I work I feel very tired. I get so much tired; moving up and down, washing, doing this and that, and in the end, I feel very tired. I feel my life becoming very weak, so that’s what really disturbs me”***(55 years old, married female)**

**“***…That would force me to stay awake all day and night, there I felt like am really tired. That forced me to feel tired and I complained that at least if all the others were here also and we share the burden. Even upon getting tired, they don’t even say that though the other one is getting tired, let’s find a way of helping her out… I get tired but there isn’t any way they are willing to help me”***(49 years old, married female)**

One caregiver mentioned that even when she falls sick, she had to care for the older person and this was so burdensome on her weak body. Another caregiver also mentioned that she had lost appetite and could no longer eat but only drink. That would make her feel very dizzy. Due to heavy workloads and poor feeding, some caregivers reported significant weight loss. One caregiver further noted that even if she tells her mother that she is tired, her mother will not understand her.

*“…I don’t eat whenever I find that she has dirtied the whole place. I was strong but I have lost weight because of that situation. I can’t eat, what I do is drink. Whenever I don’t have what to drink I feel dizzy. When am going to eat I fail because I feel I want to vomit there and then because of the tasks I do; her belongings which I always wash. It really forces me not to eat…”***(55 years old, married female)**

*“I get tired a bit, I have nothing to do even when I tell her that I am not feeling well she cannot understand me… For sure I also don’t have energy but I cannot tell her that because she will not listen because she keeps telling me to help her and take care of her needs”***65 years old, widowed female)**

Furthermore, caregivers said that they spent sleepless nights to noise made by the old person at night and this would not allow them to sleep.

“*It has affected me because I fail to get sleep when she makes noise in the night if I wake up and hear the noise I fail to get peace and I think she will disappear and I wake up to check up on her and then I get back to bed I fail to get sleep so I don’t sleep well and I end up dozing during the day”****(46 years old, widowed female)***

#### Psychological burden

The caregivers mentioned that they faced psychological burden in form of irritability from the older people with BPSD who abused them. This made the caregivers angry with the people they cared for.

“*For sure there are at times she abuses me and I get angry but the good and bad I just decided to ignore and I know that she is not in her right senses because at times she can abuse me like now and later she comes telling me good things”***(46 years old, widowed female)**

*“Whenever he gets hungry then he starts abusing me and later he asks me to forgive him. and I also tell him that I don’t have any piece of land where to cultivate sweet potatoes and I also ask him surely why are you abusing me? Thereafter we again get back to the line and we become happy. But about the issue of abusing me comes up when we get hungry; that’s where he starts abusing me”***(48 years old, married male)**

Still, caregivers faced immense opposition from older people when making decisions about the older person’s care. Caregivers lived in a state of fear for their lives since the people with BPSD turned violent and would hit the animals at home and even destroy household property.

“*The dangerous thing which I see, like me which is going to change me is how they throw tantrums…Once you tell her something trying to explain to her, she will want to oppose you immensely”***(49 years old, married female)**

*“When am at home I feel very scared. At one point she got a hoe and hit a dog and it died instantly so I fear that if she hits me one day I may also die so my life is at high risk (laughter) so I am always praying that God should help me to keep her safe and not harm anyone”***(46 years old, widowed female)**

#### Social burden

The caregivers mentioned that they face social pressure fearing that people would judge and laugh at them if they do not take good care of the patients. Due to demanding caring roles, some caregivers have resorted to remaining home and taking care of people with dementia hence social isolation. Another caregiver said that she had left her marriage to care for her old mother.

*“The behaviour she displays maybe is she does not want me to go away from home. And she does not want me to get out of home and whenever I go out and I delay a bit I will find her when she’s already not happy”***(48 years old, married male)**

*“…I left my marriage and I am at home at my mother’s home and my mother had no property so we are both needy”****(65 years*old, married female*)***

### Coping strategies

The caregivers shared different coping strategies which they used to continue caring for older people with BPSD amidst the burdens described above. The coping strategies included emotion-focused coping strategies (religious coping, acceptance and emotional support seeking).

#### Religious coping

Spiritual coping was the most predominantly used coping strategy. Most participants mentioned that they go to church and pray when they felt overburdened by the caring roles. After praying, the caregivers said that they felt relieved and motivated to continue taking care of older people with BPSD. Some caregivers revealed that believing in Christ helped them not to give up caring for older people. One of the caregivers also said that because she believed that God brought the sickness, she is ready to do God’s will of caring for her husband.

**“***I used to fear but later I kept on going to church for prayers as I entice her and she would sometimes accept to come to church. After that, I got used, so whenever I look at something coming I decide to pray and ask God to lead me through as my guide. And I realize that what we were thinking about we have gone through it”***(48 years old, married male)**

*“What helps me to manage this situation; let me tell you, is being close to religion and prayer. For I am a catholic and you know praying to Mary’s mother to intercede for us. That thing has helped me to get involved in religious matters and it has helped me so much. Otherwise, I would have given up”***(40 years old, married female)**

*“It’s a prayer that helps me. I know that it is God who brought this sickness and is the one that will take it away so its prayer that is keeping me strong”***(48 years old, married female)**

#### Persistence

The caregivers reported that they persist to keep caring for older people with BPSD. Most caregivers said that they have persistently cared for older people because they had no alternative. Some caregivers mentioned that their strong relationship (like child-parent relationship) with the older people is what motivated them to continue with caring roles. Married caregivers revealed that they must be patient to care for their partners as they promised during marriage vows to stay together in health and sickness.

*“I feel bad but I persist because I know that it’s not his wish to be like that. I will take care of him as I promised him when we got married and I wait until God decides what to do with him. I can’t even be tempted by the earthly things that may tempt me to leave him to look for other men”***(48 years, old married female)**

“*…for me, I think the situation in which am in it is all about persisting and I pray to God such that He gives me strength to take care of the old woman as I also survive”***(48 years old, married male)**

#### Emotional and social support

Emotional and social support was sought from religious leaders, friends and family members. The caregivers reported receiving emotional and social support in form of counselling, encouragement and companionship. One caregiver mentioned that he was having distressing thoughts and had even lost weight but when he received counselling, he normalized.

*“…It is because of going to church or getting religious people to come home and they counsel me. This makes me feel better”***(46 years old, widowed female)***“…those who encourage us they are the members of the church, they also come and encourage us just like that…Even friends they come and encourage them, spend the whole day with them, like how I have told you that they are old, they like talking” (****49 years*old, married female*)***

*“I even changed my moods and lost weight because I was thinking a lot. But what helped me, I met with a group of counsellors. They had to tell me that since I have taken up that responsibility, stop thinking about any other thing and focus. So that challenge where I was almost developing hypertension really would disturb me a lot. But after I had counselling services, I had to normalize again…That is socialization; being social with brothers and sisters and even neighbours. That has been the greatest factor”****(52 years*old, married male*)***

#### Planning

The caregivers set plans to enable them to adequately care for older people with BPSD. Some caregivers mentioned that they planned for their time according to their caring needs.

“*So, the way how I take care of her; it’s like I gave her a timetable and so far she’s used it. She knows that early in the morning, she’s supposed to wake up and bathe. After bathing, she takes her breakfast, after breakfast, she will sit at her place”***(52 years old, married male)**

In addition, one caregiver revealed that he joined money-saving groups to keep money that can be used in future when there is a need. Some caregivers said that they have engaged in farming to produce food for the family. Another caregiver mentioned that she is training and mentoring her children such that they will be able to take care of their grandparents when she can no longer do it herself.

*“Me, my ability, of course, I plan for my future. Like now, I try to invest or trying to look for the available resources and they bring in money. Let’s say I like farming. And it’s not that I go there and dig, you will know that in this place we grow Irish potatoes and I like them, I usually get money out of it and even beans…And another thing, I invest for the future by joining saving groups where we pool for one another and like now they pool it for me and I take it to the bank account. So it cannot happen that you find my account with no money for emergency”***(40 years old, married female)**

*“So far where I have reached, am starting to involve my children. Such that in case I have started to become weak, they can also take care of their grandparents”***(38 years old, married male)**

#### Acceptance

The caregivers were resilient towards their caregiving roles. One of the caregivers said that there is no one else who is willing to take care of the old woman and so she has to accept and some viewed caregiving as a God-given role.

*“So, if I get tired of her and I send her to the daughter-in-law, will she be the one to accept her yet she’s not her biological daughter? So, in that case, I must accept”***(38 years old, married female)**

*“So, it’s all about accepting. I accepted and once you go to religion, they will tell you that it is a gift which God gave to me such that maybe I take on caregiving. So, am contented per now, am contented to look after her until when she dies or I die”***(40 years old, married female)**

#### Self-distraction

Some caregivers mentioned that they use self-distraction to forget about the challenges of caregiving. One participant noted that she sings to get her mind off the daily routine and therefore forgets about the stress of caring.

*“I try to keep myself busy, sing and seem as if I have forgotten about that. Whenever I sing, I feel as if I have forgotten about it”***(55 years old, married female)**

## Discussion

We aimed to explore the caregiver burden and coping strategies used when caring for older people with BPSD in Rubanda and Rukiga districts in Southwestern Uganda. We found that caregivers’ burden was due to factors such as financial, physical and psychological burdens. We also found that most caregivers used emotion-focused coping strategies such as religious coping, acceptance and emotional support seeking to cope with stress resulting from the caregiving burden.

### Caregiving burden when caring for older people with BPSD

The caregiver burden is clinically significant and steadily rising among caregivers of people with dementia [22]. In this study, we found that caregivers’ burden was due to other factors such as financial, physical and psychological burdens. Similar findings were reported by a study in India[23]. Unlike in our study in which financial burden was the most reported cause of distress to caregivers, a recent study in Kenya reported that emotional distress was the commonest form of caregiver burden [24]. This inconsistency between these 2 studies may lie in the good quality of healthcare systems in Kenya, that lessens the financial burden. Whereas Ugandan caregivers of people with dementia may experience both emotional and financial burdens, their financial burden may stand out due to its inherent modulating effect on the emotional burden [25]. This financial burden was mainly due to unstable sources of income, increasing caregiving expenses and lack of time for working. These findings are consistent with a Kenyan study which found that caregivers quit their jobs to stay home and do full-time caregiving for older people with BPSD [24]. Therefore, financially sustaining the care of people with BPSD competes with other family needs.

The physical burden was significant due to exhausting caregiving roles. The psychological burden was due to the irritability of the older people who abused caregivers. These findings are consistent with those of another study among caregivers of people with dementia in rural Uganda [26]. Given the potentially overwhelming caregiving burden, there is an urgent need to put up measures which can be sustainably used to improve caregivers’ well-being and thus enhance their caregiving capacity.

### Coping strategies when caring for older people with BPSD

Caregivers respond differently to caregiving burdens and they adopt different coping strategies. We found out that most caregivers used religious coping whereby they consistently prayed and believed that God will help them to go through their challenges. Other coping strategies used by caregivers were acceptance, planning, seeking emotional support and self-distraction. Unlike a previous study by Ainamani et al., 2020, we did not find substance misuse as one of the coping strategies used by caregivers.

In this study, we found that most caregivers use emotion-focused coping strategies like religious coping, acceptance and emotional support seeking to cope with stress resulting from the caregiving burden. Similar findings were reported by a previous study in Brazil [27]. The caregivers received prayers and counselling from religious leaders and counsellors. Spirituality and religious beliefs are associated with the mental health and well-being of caregivers [28].

Previous studies have linked emotion-focused coping with better outcomes on the mental health of caregivers [17]. Conversely, previous authors have strongly associated problem-focused coping with lower caregiver burden severity. They have reported a significant correlation between problem-focused coping and the psychological well-being of caregivers [29]. However, there is still no clear process to decide which coping strategies are good or bad. The choice and effectiveness of a coping strategy are dependent on individual characteristics, values, beliefs and the stressor at hand [29]. Therefore, future research efforts should be focused on context-specific coping strategies for caregivers of older people with BPSD to facilitate their coping process with their caregiving role.

In addition, acceptance and persistence were noted to be common coping ways. This was based on interpersonal attachments between the patient and the caregiver for example marital, sibling or child-parent relationships. The relationship and attachment between the caregiver and the patient may act as a driving force for the caregiver to continue the caregiving role [30].

### Study limitations and strengths

In this study, there were methodological shortfalls that are worth mentioning. To start with, our data was collected in the local language (Runyankole-Rukiga) and then transcribed and translated into English. In this process, participant responses could have been altered. We did not independently confirm the diagnosis of dementia, thus some of the people being cared for could be not having dementia. Lastly, our findings could have been affected by participant bias due to strong social and cultural values held in the area of study forcing participants to give socially and culturally acceptable to give responses about coping in particular.

The strength of this study was that we used a triangulated data collection process. We included individuals from two communities to gain diverse perspectives of burden of care and coping strategies of informal caregivers of people with BPSD.

## Conclusion

This study highlights that caring for people with BPSD is a demanding role with social, physical, psychological and financial strain on the caregiver. Despite the burden of caring for people with BPSD, caregivers adopted problem-focused and emotional coping strategies to alleviate the impact of the burden of care. Important there is need for policy makers at all levels to direct efforts towards improvement of the health care system to address the needs of people with BPSD and their families. More research needs to be carried out to develop socially and culturally appropriate family based interventions to support people with BPSD and their caregivers.

## Data Availability

Full datasets related to this study are not publicly available to maintain the privacy of the individuals interviewed during this study. However, de-identified data can be available from the corresponding author upon reasonable request.

## List of abbreviations

ADRDs: Alzheimer’s disease and related dementias
BPSD: Behavioral and Psychological Symptoms of Dementia
ROTOM: Reach One Touch One Ministries
VHTs: Village health teams
